# Dose verification of helical tomotherapy intensity modulated radiation therapy planning using 2D-array ion chambers

**DOI:** 10.2349/biij.6.2.e24

**Published:** 2010-04-01

**Authors:** S Xu, C Xie, Z Ju, X Dai, H Gong, L Wang, J Yang

**Affiliations:** 1 Department of Radiation Oncology, PLA General Hospital, Beijing, China; 2 Department of Radiation Oncology, Monmouth Medical Center, New Jersey, United States

**Keywords:** Tomotherapy, dose verification, IMRT, radiation therapy, QA

## Abstract

**Purpose:**

To investigate the clinical usage of dose verification of Helical Tomotherapy plans by using 2D-array ion chambers, and to develop an efficient way to validate the dose delivered for the patients during treatments.

**Materials and Methods:**

A pixel-segmented ionisation chamber device, IMRT MatriXX™ and Multicube™ phantom from IBA were used on ten selected Tomotherapy IMRT/IGRT head and neck plans in this study. The combined phantom was set up to measure the dose distribution from coronal and sagittal planes. The setup of phantom was guided for verifying the correction position by pre-treatment Tomotherapy MVCT images. After the irradiation, the measured dose distributions of coronal and sagittal planes were compared with those from calculation by the planning system for cross verification. The results were evaluated by the absolute and relative doses as well as Gamma (γ) function. The feasibility of the different measuring methods was studied for this rotational treatment technique.

**Results:**

The dose distributions measured by the MatriXX 2D array were in good agreements with plans calculated by Tomotherapy planning system. The discrepancy between the measured dose and predicted dose in the selected points was within ±3%. In the comparison of the pixel-segmented ionisation chamber versus treatment planning system using the 3 mm/3% γ-function criteria, the mean passing rates of 2 mm dose grid with γ-parameter ≤1 were 97.37% and 96.91%, in two orthogonal planes (coronal and sagittal directions), respectively.

**Conclusion:**

MatriXX with Multicube is a new system created for rotational delivery quality assurance (QA) and found to be reliable to measure both absolute dose and relative dose distributions, simultaneously. It achieves the goal of an efficient and accurate dosimetry validation method of the helical delivery pattern for the Helical Tomotherapy IMRT planning.

## INTRODUCTION

With the development and combined utilisation of Intensity Modulated Radiation Therapy (IMRT) and Image-Guided Radiation Therapy (IGRT) for the past few years, forms of Helical Tomotherapy and advanced Intensity Modulated Arc Therapy (IMAT) have been evolving into new kinds of rotating radiation treatment techniques after step-and-shoot and sliding window IMRT deliveries. With the improvement of the beam entry angles and modulation parameters, it is obvious that rotational therapy will become an important delivery methodology in modern radiation oncology departments for better dose conformality and critical organ sparing. One of the important characteristics of rotating radiation is its dynamic nature and dosimetry variability in radiation delivery. Therefore, this is a great challenge for clinical physicists currently seeking tools to achieve quality assurance (QA) goals of this dynamic treatment planning and its associated delivery issues.

Radiochromic film has been proven as a viable tool for quantitative 2D dosimetry for conventional and IMRT modalities for quite some time [1-13]. The greatest advantage of film dosimetry is its fine spatial resolution. However, film dosimetry is somewhat influenced by the limiting factors which are time-consuming, uncertainties of developing-chemicals, and tedious analysing process. Pre-treatment patient plan verification is a part of standard procedures for IMRT treatment. No adequate solution has been available for the new dynamic techniques such as IMAT and Tomotherapy. Point-measurement with ionisation chambers is usually insufficient and film dosimetry requires a lot of work if clinical physicists try to achieve excellent agreements. Vendors who have provided the 2D array detectors are all engaged in the new development for the verification of rotational dosimetry. Sun Nuclear has produced the MapPHAN™ along with its MapCHECK™ diode array to serve this purpose in acquiring the dose verification. Initial results are promising from multiple-angle testing. However, for lateral-entry beam angles, the phantom material buildup with detector location creates certain levels of dosimetric uncertainties. If a proper phantom material calibration is applied, then the dosimetry data are quite comparable to the EBT film dosimetry provided by International Specialty Products (Wayne, NJ, U.S.A.). With the new design specification, the rotational-treatment delivery verification using a 2D matrix detector to acquire rotational dosimetry data was described in detail [14]. PTW has also introduced the OCTAVIUS™ phantom for the QA of rotational treatment, along with their 2D-array ion chamber matrix which is currently being used in the planar dose analysis. They also claimed that the signal acquired is independent of the beam angles [15]. A novel 3D device Delta-4 (ScandiDos, Uppsaka, Sweden) has also been reported in acquiring the 3D dose maps for IMRT treatments, which claimed to be useful in the rotational dosimetry measurements.

Herzen J et al. reported that the response of a MatriXX QA device is linear with dose and energy independent. The authors concluded that the detector is a suitable device for QA and 2D dose verifications for IMRT QA [16]. The same QA device has also been investigated for proton therapy QA by the group from MD Anderson Cancer Center [17]. Their MatriXX measurement results on different energies of proton therapy beams were with the accuracy comparable to those of ion chamber measurements and film dosimetry. More recently, Li JG *et al*. compared the two commercially available detector arrays (MapCHECK^TM^ and MatriXX) for their use in the QA of patient-specific IMRT treatment plans [18]. Both detectors showed negligible errors (< 1%) when measuring doses of more than ˜8 cGy, but exhibited errors of ˜3% when measuring doses on the order of 1 cGy. The authors obtained excellent passing rates for both detector arrays when compared with the planar dose distributions from the treatment planning system for three 6 MV IMRT fields and three 18 MV IMRT fields. For the verification of rotational delivery, such as Tomotherapy or volumetric modulated arc therapy, the MapPHAN™ was developed for new or existing MapCHECK users.

In this article, the authors utilised the newest development, MatriXX phantom with the 2D ion chamber arrays, from IBA (Louvain-Ia-Neuve, Belgium) to evaluate the most difficult head and neck treatment cases at the authors' center. The methodology mentioned in this article was different from the conventional QA verification procedure which only achieved the fixed beam entry angle of LINAC with planar dose distribution [19]. This particular verification system is created to assess 2D array suitability for rotational dosimetry. This QA study was performed for ten-patient plans from Helical Tomotherapy, with proper buildup and geometry for side scattered compensation. This 2D MatriXX device then transformed into a pseudo 3D tool for various plan verification in order to conquer the complex helical nature of radiation delivery.

## MATERIALS AND METHODS

This QA phantom composed of MatriXX and Multicube was positioned on the treatment couch, verified with isocenter alignment tool provided by Tomotherapy planning and the laser systems. The irradiation source used for verification process is a single energy photon beam of 6 MV on a linear waveguide. MatriXX device consists of 1020 (32x32) vented pixel ion chambers. Each chamber occupies 0.08 cm^3^ volume and holds the resolution of 7.62 mm. If multiple readings are generated, then 1 mm resolution with interpolation is applicable. Measurement dose rate ranges from 0.1 to 20 Gy/min with the dose resolution of 0.5 mGy/min. All verification plans in this study were created for this phantom on Tomotherapy planning station, with version 2.2.4. Measurement software provided by IBA dosimetry is OmniPro-IMRT version 1.6.

### Acquisition of the phantom images for verification

**Figure 1:**
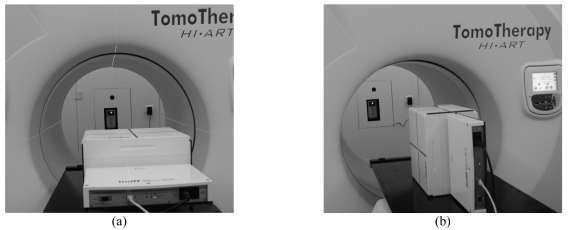
Pictures of IMRT MatriXX 2-D Array and Multicube phantom clinical setting used to measure the Tomotherapy IMRT plan from two orthogonal orientations – (a) coronal, and (b) sagittal – respectively.

The pixel ionisation chamber MatriXX was inserted into the Multicube phantom in order to form a complete assembly with a proper buildup, similar to the setup layout for dose verification picture on the couch of Tomotherapy system as shown in Figures 1a and 1b. The assembly images for coronal and sagittal orientations of the combined phantom were previously scanned by a Philips Brilliance Big Bore CT simulator (Philips Medical Systems) and stored as a phantom data set. A 2 mm slice thickness was used to guarantee the precision of 2D dose distribution calculated by Tomotherapy planning system, and also to maintain the exported intensity map iso-tropically for alignment purpose. The suitable locations to place the three fiducial marks were the pre-designed crosshairs on the MatriXX’s enclosures. This helps easily identify the center of the device on the phantom images acquired by the CT scanner. While generating the CT DICOM data set, the user has to make sure the phantom images are named accordingly for proper patient-specific identification. After the proper configuration and entry of basic information, images of combined phantom along coronal and sagittal orientations were then imported into Tomotherapy planning station for calculation of all patients in this study.

### Dose Verification

#### Tomotherapy Delivery QA (DQA) setup

First, after importing the pre-scanned CT images of the combined phantom for the corresponding setup, the image value-to-density table (IVDT) for the dose converting parameters is imported simultaneously for dose calculation. Secondly, the CT couch was removed from the phantom images and was replaced by the Tomotherapy delivery couch so that the different geometry effect of the couch was accurately modeled during the dose calculation process. According to the patient image volume, register the phantom images and save the laser positions to the DQA plan for setup correlation. Isocenter location is then properly determined. At the end, the dose distribution was calculated for the phantom image volume for each specific patient based on the final calculated and deliverable plan. The dose intent of the phantom planning should be identical as the prescribed dose to the patient per fraction from the final approved plan.

#### Systematic measurement preparation

Warm-up time of MatriXX 2D-Array ion chamber usually takes at least 15 minutes, as it is required before true delivery starts. Similar to other 2D-Array ion chamber devices available for clinical usage, a total dose of 100 cGy was pre-irradiated on the MatriXX array in order to eliminate noises and to keep the uniform dose response characteristic with better signal-to-noise range. This pre-irradiation procedure of IBA MatriXX array specially created for Tomotherapy system was automatically moving the couch longitudinally into the bore while maintaining 5x40 cm MLC opening at the static position at 0 degree. This method will keep the 2D-Array ion chamber irradiated uniformly with minimum noise differentiation of the delivery dose responses. For the absolute dose calibration, there are two possible ways to perform this task. A typical calibration field was delivered at the dmax point to cross calibrate the central ion chamber, which represents 1 cGy/MU with the C-arm LINAC. This method can be performed with a conventional LINAC on site and cross comparison has to be executed in measuring the Tomotherapy calibrated dose. Another method is to use the Tomotherapy static QA procedures to deliver known doses to the associated ion chamber locations, thus obtaining the corresponding calibration factors to the specific ion chamber (at least the center ion chamber needs to be cross checked).

**Figure 3:**
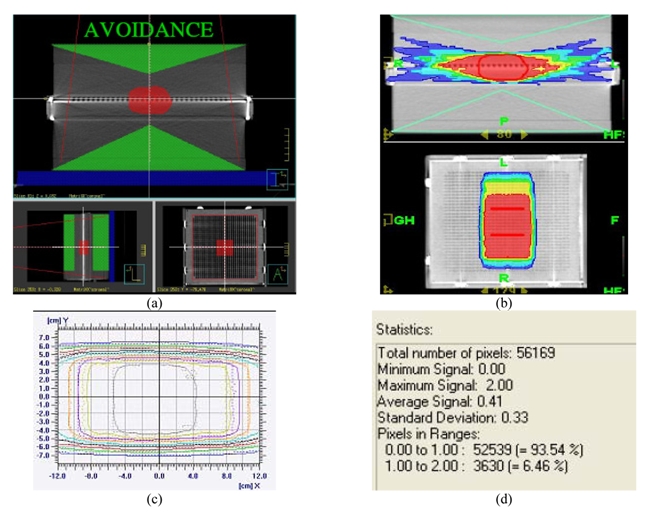
MatriXX directional sensitivity planning and measurement validation. (a) Target contours, (b) Planned, (c) Planned and measured isodose lines, (d) Statistics [20].

According to the experiments performed by Tim Holmes, Ph.D. [20], the directional dependence of this MatriXX system is quite acceptable for rotational delivery. The 1020 ion chambers are configured in a way that very little high Z metal is present in the radiation field and the calculation is very accurate (convolution/superposition dose model). Rotational delivery can tolerate relatively larger errors over small angles. With 51 beam entries, even a single lateral beam contributes 10% dose error, which will only translate into 0.2% total dose error. Geometrical blurring is also inherited in the rotational delivery, hence errors occurring over small distances are blurring out. An anterior avoidance plan has been delivered to verify the lateral beam angle dependence by Tomotherapy and MatriXX - results indicated that the passing rate of a lateral beam entrance plan is 93.54%, which has proven this device has minimum lateral beam entrance effect in Tomotherapy delivery. (Figure 3)

#### Verification measurement for Tomotherapy IMRT plan

The phantom plans for ten randomly selected patients with the coronal and sagittal directions were designed and saved into the data server for validation. These selected patients are all Nasopharyngeal Carcinoma (NPC) patients with patient 9 and 10 being the most challenging cases with a sharp dose gradient through the cord, brainstem and parotid glands while the tumor location is critically located in between. The reason for selecting head and neck cases is because such cases consist of the best testing modality for the rigidity of MatriXX for QA. Also, head and neck cancers are often very difficult to deal with. Another reason is that head and neck cases are the most common cancers in the authors’ areas which need IMRT treatments, and also present a tough choice for QA methodology. Before the delivery of the DQA plans, the set-up positioning as well as the laser alignment configuration are demonstrated as Figures 1a and 1b. Set-up accuracy of combined phantom location was corrected and confirmed by using planning CT/MVCT registration process, which is a standard procedure at the Tomotherapy operational console. During the pre-treatment IGRT process, Multicube and BBs are the areas that need to be covered by the MVCT scan, typically we use fine scan for the alignment purposes. If the laser and couch sag are properly managed, then the manual shift is not necessary because the auto-registration of Tomotherapy program functions well with MVCT images of MatriXX and its associated CT scans. After finishing the MVCT image registration, MatriXX within the combined phantom was irradiated according to verification plans generated from each selected patient in Tomotherapy planning console.

### Data analysis and processing after image and dose acquisition

Two methods were implemented separately in order to analyze and compare the measurement data after Tomotherapy delivery. The dose distributions measured by the 2D-Array ion chamber were compared with those calculated by Tomotherapy DQA plans using vendor provided software tools.

#### OmniPro-IMRT analysis software (from IBA)

After data is exported from the Tomotherapy planning station, the intensity map file of dose calculation results was imported into OmniPro-IMRT software and co-registered with the measured dose intensity map. The proper file to send from Tomotherapy for export into OmniPro-IMRT software is the Text DQA Header and Image Files. These are files created by Tomotherapy for export. Those two files usually can be transferred via network or even a simple USB drive to the MatriXX computer with installed analysis program. After completion of file transfers, these two files were compared by using 2 mm and 1 mm calculation grid points. 3 mm/3% DTA and absolute dosimetry errors, as Gamma (γ) criteria [21, 22] are also used for evaluating the dose distribution on both the coronal and sagittal directional planes. The results of the quantitative and qualitative analysis were compared simultaneously for the specific phantom setup.

#### Tomotherapy DQA software (from Tomotherapy)

Tomotherapy planning tool also provides the evaluation software for dosimetry analysis. While the TIF image with registration points designed specifically for Tomotherapy was exported from MatriXX data, the linear CAL file for film calibration would be given automatically. After the acquisition process of MatriXX data, one can export the TIF file with registration points designed specifically for Tomotherapy. This will deliver the TIF image and a linear CAL file for film calibration. This particular functionality in OmniPro-IMRT version 1.6 software is located under the “Export files” tab. The procedure for registering the TIF image in the DQA workspace was the same as using for a regular film-based dosimetry. The comparison between measured and calculated dose distributions was also done by the 3 mm/3% γ-criteria, which calculated the gamma histogram to display those associated parameters in a real-time mode.

## RESULTS

**Figure 2:**
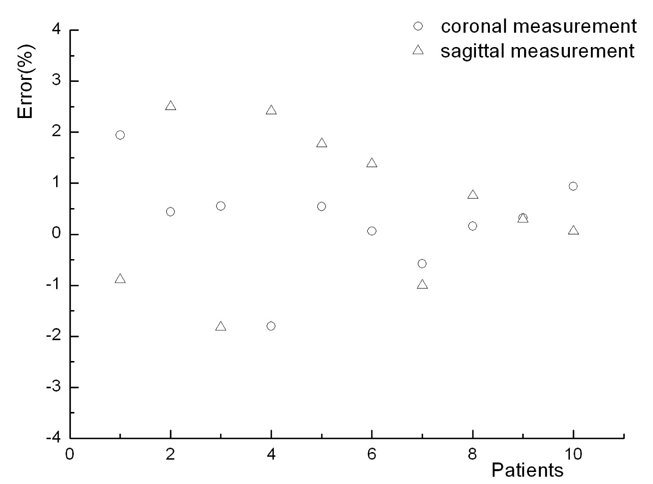
Percentage errors for the absolute dose of pre-selected Tomotherapy IMRT plans.

Dose verifications for ten pre-selected Tomotherapy NPC IMRT plans were performed in both coronal and sagittal orientations using the 2D MatriXX ion chamber device. Dose distribution between measured and calculated dose distribution showed good agreements from the data analysis. Figure 2 shows the percentage deviation of absolute dose distribution of the ten previously treated patients, which is shown within ±3% error margin. Selection criteria of points for absolute dose measurement was situated in the high dose and low gradient region of MatriXX measurement plane, according to the IMRT measurement guidelines in the AAPM Task Group report #119.

### OmniPro-IMRT software process

**Figure 4:**
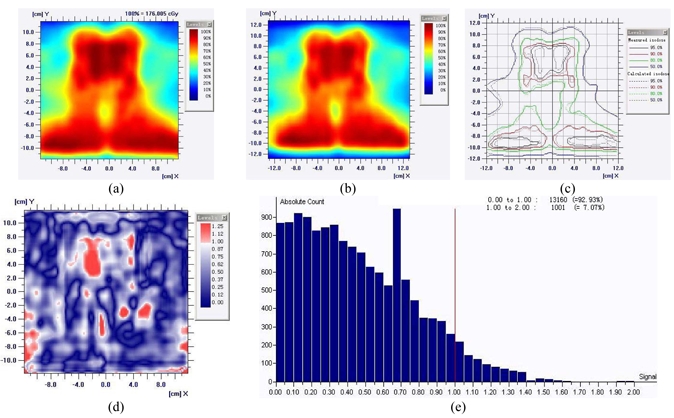
Analysis results between the measured and calculated dose at the 2 mm grid along the coronal direction. (a) Measured dose, (b) Calculated dose, (c) Dose comparison, (d) Gamma plot, (e) Gamma analysis.

**Figure 5:**
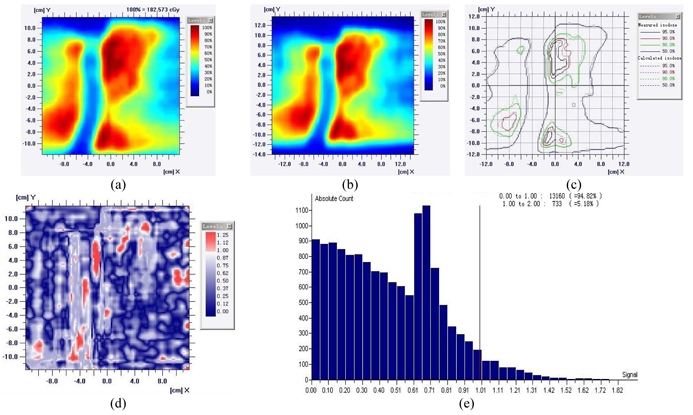
Analysis results between the measured and calculated dose at the 2 mm grid along the sagittal direction. (a) Measured dose, (b) Calculated dose, (c) Dose comparison, (d) Gamma plot, (e) Gamma analysis.

Table 1 lists the results of Gamma analysis (3%/3 mm) with 2 mm and 1 mm calculation grids, respectively, for the selected 10 NPC patients. The average passing rates for the DTA of 3%/3 mm for the coronal plane at 2 mm and 1 mm grids are 97.37% and 98.71%, respectively. And those for the sagittal plane are 96.91% and 97.86%, respectively. Figures 4(a)-4(e) and 5(a)-5(e) show the results of calculated and measured dose distributions and Gamma analysis (3%/3 mm) for a typical NPC plan along the coronal and sagittal direction. In Figure 5, the same NPC patient shown in Figure 4 is selected for analysis with the different plane (sagittal). The isodose matches and Gamma plot were displayed, as well as the statistical analysis. The passing rate is more than 90% with great confidence level of clinical implementation.

### Tomotherapy DQA analysis

**Figure 6:**
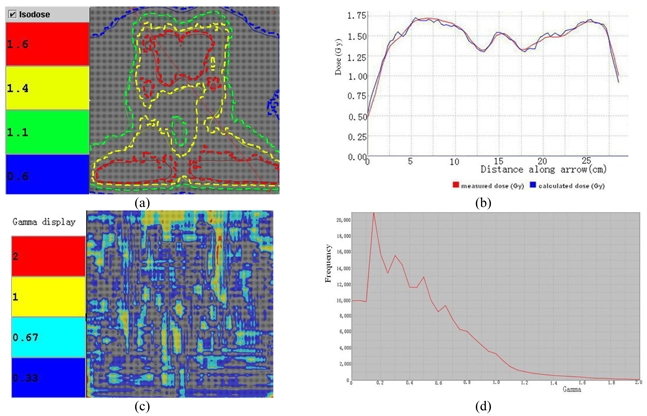
Comparison of measured and calculated results on the coronal measurement of MatriXX. (a), (b), (c) show the profile distribution and the Gamma results for the plan along the coronal setup (Isodose curve comparison, dose profile of the arbitrary surface, and gamma plot respectively). 6(d) is the Gamma histogram.

**Figure 7:**
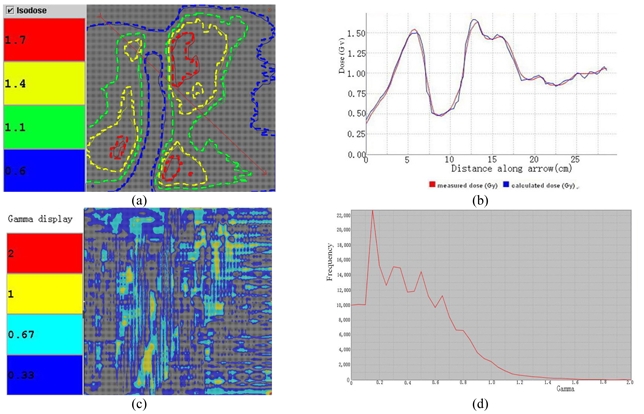
Comparison of measured and calculated results on the sagittal measurement of Tomotherapy provided software. (a), (b), (c) show the profile distribution and the Gamma results for the plan along the saggital setup (Isodose curve comparison, dose profile of the arbitrary surface, and gamma plot respectively). 6(d) is the Gamma histogram.

Although the Tomotherapy dosimetry analysis software does not provide passing rates for the dose distribution, in the Tomotherapy DQA process, dose fluence (profile) and histogram of the combined 3% and 3 mm gamma criteria could still be analysed. Figures 6(a)-6(c) and 7(a)-7(c) show the dose distribution and the Gamma results for the plan along the coronal and sagittal setup direction. And 6(d) and 7(d) show the Gamma histograms, with two directional setups.

## DISCUSSION

Clinical example in Figure 4 shows a typical NPC patient with bilateral PTV coverage and the critical component for IMRT is cord sparing. Coronal planned/measured dose comparison using MatriXX and Multicube has shown excellent agreement, with passing rate 92.93% of 2 mm calculation grid. Gamma index plot also indicates only few areas are larger than 1.0. Figure 5 shows the sagittal view of the same patient MatriXX evaluation; however, the passing rate has increased to 94.82% with same dose grid calculation. The cord sparing of the measurement is very obvious and Gamma plot shows only few areas with the index larger than 1.0. Figures 6 and 7 are the reports generated from Tomotherapy evaluation tools with similar passing rates and the Gamma index plot, which has confirmed the QA device of MatriXX can be an excellent substitute of the film dosimetry due to the similar experimental results.

The grid for dose calculation within Tomotherapy phantom plan was set at 2 mm and it was the same resolution as in the phantom planning and fan-beam CT scans. The 2 mm calculation grid was set in such a way that the longitudinal direction can match the CT scanning slices. Regarding the selection for the find grid for the dose calculation on the axial slice, the field of view is set at 512x512 to reach the maximum image resolution for calculation. In order to achieve the patient dose verification before the patient’ delivery, 2 mm on the calculation grid should be also used for registration purpose. Even though the passing rate with 1 mm grid was slightly higher than that with 2 mm, it would be better to select the 2 mm precision of dose calculation in these plans.

Improvement of the MatriXX efficiency, compared to the regular Tomotherapy DQA procedures, the QA time has been cut down from 90 minutes film dosimetry to about 43 minutes for each patient DQA using MatriXX. Major time saving is from no marking and processing the film for measurement. Also, ion chamber of MatriXX reports the dose value immediately if properly calibrated, no separate ion chamber measurement setup is needed for point dose measurement. Tomotherapy represents a new approach of dynamic rotational IMRT with excellent image guided capability. Dose verification has been achieved by using the MatriXX 2-D array phantom along the coronal and sagittal directions for ten specific Tomotherapy patients. The analysis results for the absolute and relative doses between measured and calculated were found in excellent agreements. Therefore, the MatriXX is a 2D QA device, not only for dose verification of dynamic IMRT on conventional LINAC [23, 24], but also for helical Tomotherapy IMRT/IGRT treatment validation. Results of the measurement for two different setups have shown that the MatriXX on both directions are capable of generating superior clinical dosimetry results and extremely effective for measurements in helical Tomotherapy. The number of passing points with γ-parameter ≤1 in coronal and sagittal direction also show excellent agreements with the final approved plans. Even with the tight margin and very complicated NPC cases, the authors still observed more than 90% passing rate. For instance, if the combined 5%/3 mm criterion was used for analysis, the number of points with γ≤1 would increase to 94.37%. In addition, these results from two different analysis software (IBA and Tomotherapy) were very consistent. But if Tomotherapy DQA software is being utilised for the analysis, the counter clockwise setup along sagittal direction of the combined MatriXX phantom is recommended strongly for a better passing rate (Figure 1(b)).

Chan et al. also reported that the diode array data showed a trend of angular dependence, decreasing from 100% at 0º down to 90% at 80º gantry angle [25]; however, the overall measurement results from rotational delivery are within clinically acceptable accuracy due to the averaging effect from all the gantry angles [26]. Rotational delivery usually can tolerate larger errors in smaller angles, and the authors have observed the directional sensitivity of MatriXX ion chambers with water equivalent Multicube to less effect in the Tomotherapy delivery scheme. The MatriXX in Multicube phantom is ion chamber array system and it is well known that the angular dependence of ion chambers is not as severe clinically for rotational delivery. Planar dose validation device with buildup certainly presents the great challenge and that is the reason for SunNuclear to create a device called ArcCHECK™ to truly serve the arc therapy dosimetry QA purpose. This is one of the first reports on patient-specific rotational dosimetry verification using MatriXX and Multicube.

However, compared to the traditional film dosimetry, MatriXX is still a much easier device in clinics than the film-based QA program. In this study, the authors selected 10 NPC patients due to their sharp dose gradient and complexity, which bear the challenges to acquire superior agreement compared with simple prostate IMRT cases. This patient pool represents a challenging situation and the authors also observed good agreement with high passing rates in both coronal and sagittal planes.

Similar to other commercial 2D-array, the effective field-size of 24.4×24.4 cm^2^ of the MatriXX system presents some limitations (i.e., QA on large IMRT fields). But it should be a key issue for dose verification on most Tomotherapy IMRT plans. However, it's important to avoid irradiation of the electronic parts outside ion chamber so that it will not affect the lifetime of the QA device. On the other hand, the spatial resolution of MatriXX due to its structure is less than those of film for dose verification as obviously noticed. The area of high-dose gradient should be further considered for data analysis. From the final study results, threading effect was inevitable in the helical Tomotherapy delivery process. With the development of hardware and software of commercial 2D-Array, it’s possible that dose verification using 2D-Array ion chamber could provide the cubic rotational dose reconstruction accurately. Weight of the combination of MatriXX and Multicube is another concern, the total weight is about 33 kg, which could also be cumbersome in transporting the phantom to and from the couch top.

## CONCLUSION

MatriXX 2D-Array has provided a simple and effective dose verification tool for rotational dynamic IMRT such as Tomotherapy technique. The measurement for the final plans of Tomotherapy shows that MatriXX is capable of both absolute and relative dose measurements within good agreements. Its geometrical-machine buildup for the 2D array reduces uncertainties during the helical delivery in comparison with the Tomotherapy DQA dosimetry modules. MatriXX with Multicube phantom creates a smoother and efficient operation with reasonable QA analysis time and results for helical Tomotherapy at the authors’ busy cancer center.
